# Stroke volume variation remains accurate in the presence of proximal stenosis

**DOI:** 10.1186/s40981-024-00693-5

**Published:** 2024-02-13

**Authors:** Hiroatsu Sakamoto, Atsuhiro Kitaura, Shota Tsukimoto, Yukari Yoshino, Takashi Mino, Haruyuki Yuasa, Yasufumi Nakajima

**Affiliations:** 1 Department of Anesthesiology, Faculty of Medicine, Kindai University, 377-2 Ohno-Higashi, Osakasayama, Osaka 589-8511 Japan; 2 Department of Anesthesiology, Kanagawa Dental University, 82 Inaoka, Yokosuka, Kanagawa 238-8580 Japan

Arterial pressure line insertion is common practice for perioperative management and to manage infusion using dynamic indicators [1]. Although arterial pressure line information may be influenced by the vessel in which the line is inserted [2, 3], this aspect is not commonly considered. If there is a proximal artery stenosis of the arterial pressure line, the information obtained from that line may be isolated from systemic circulatory dynamics. However, no actual clinical data have been reported. Therefore, we report the case of a patient with vascular stenosis in the left subclavian artery who underwent bilateral radial arterial pressure line placement and measurements of which were compared over time. A 71-year-old woman with hypertension received radical esophagectomy for esophageal cancer. During surgery, an aortic injury was caused by an auto-anastomotic device (yellow arrow in Fig. 1) during resection of the anal-side fragment of her esophagus at the end of the esophagectomy. Emergency endovascular aortic repair was performed immediately. After stent graft insertion, we found the large pressure gradients in the upper extremity and femoral artery. As a result, an additional left axilla-femoral bypass (green arrow in Fig. 1) was performed. Bilateral chest tubes were inserted during her surgery. After intensive care unit (ICU) admission, the patient was deeply sedated with 50 µg/h fentanyl, 0.4 µg/kg/h dexmedetomidine, and 3 mg/kg/h propofol, with ventilation (pressure control/assist control, inspiration pressure,14 cmH_2_O (tidal volume was approximately 7 mL/kg); frequency, 12/min; fraction of inspiratory oxygen, 0.5; positive end-expiratory pressure, 5 cmH_2_O). Physical inspection at the time of ICU admission revealed an arterial blood pressure difference of 50 mmHg between the two upper limbs. The postoperative CT scan revealed the pressure difference was caused by stenosis of the left subclavian artery-prosthetic anastomosis (red arrow in Fig. 1). Therefore, an additional FloTrac™/Vigileo™ device (Edwards Lifescience LLC, Irvine, CA, USA) was placed in the right radial artery in the ICU as an additional arterial pressure observation line, with fluid infusion performed as necessary. The parameters obtained from the waveforms of both radial arteries were recorded every 2 h while the patient was almost completely mechanically ventilated. During data collection, the patient was in sinus rhythm. Despite different stroke volumes (SV) between the right and left arteries, the stroke volume variation (SVV) remained almost identical, with similar responses to infusion (Fig. 2). The FloTrac™/Vigileo™ device (fourth generation) is calculated as the product of the numerical value (*χ*) derived from the algorithm reflecting vascular tone in the SV measurement, and the standard deviation (SD) as obtained from the arterial pressure waveform. SVV = 2(SV_max_ − SV_min_)/(SV_max_ + SV_min_) = 2(χSD_max_ − χSD_min_)/(χSD_max_ + χSD_min_) = 2(SD_max_ − SD_min_)/(SD_max_ + SD_min_). Although upstream vascular resistance is thought to influence *χ* and SD, its influence is excluded from the equation. Therefore, the SVV measured at the stenotic side is considered to reflect changes in circulating blood volume. It is possible that the data reported here reinforce the reliability of SVV from an arterial pressure line inserted regardless of the artery. It is rare to obtain clinical data as well suited as this case.

**Fig. 1:**
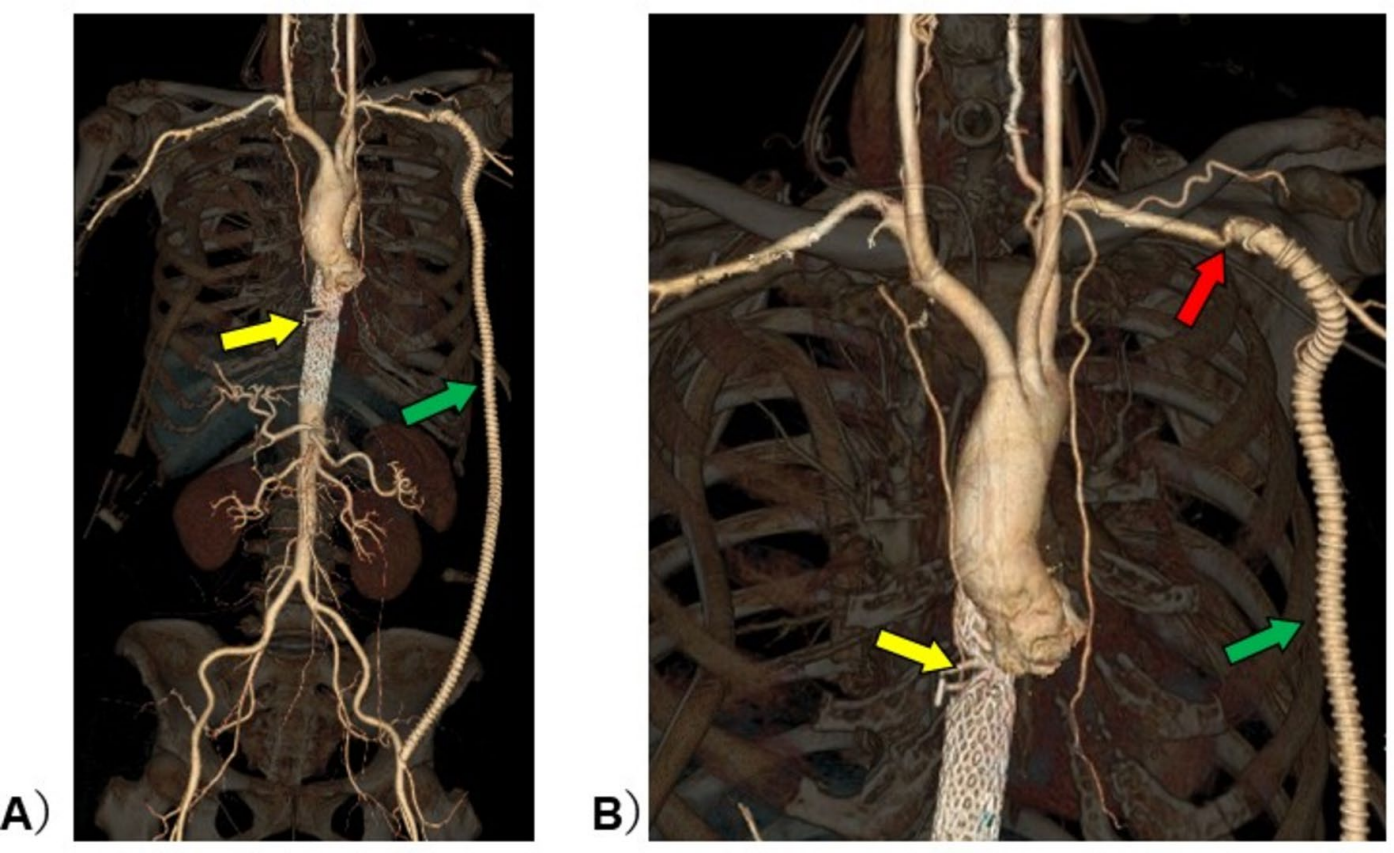
Computer tomography imaging of the patient. **A** Panoramic view of major thoracoabdominal arteries. **B** Enlargement around vascular lesions. Vessel deformation due to vascular injury and lined stent graft in descending aorta (yellow arrow). Left subclavian-femoral artery bypass with artificial graft (green arrow). Stenosis of the left subclavian artery due to distortion caused by artificial vascular anastomosis (red arrow)

**Fig. 2:**
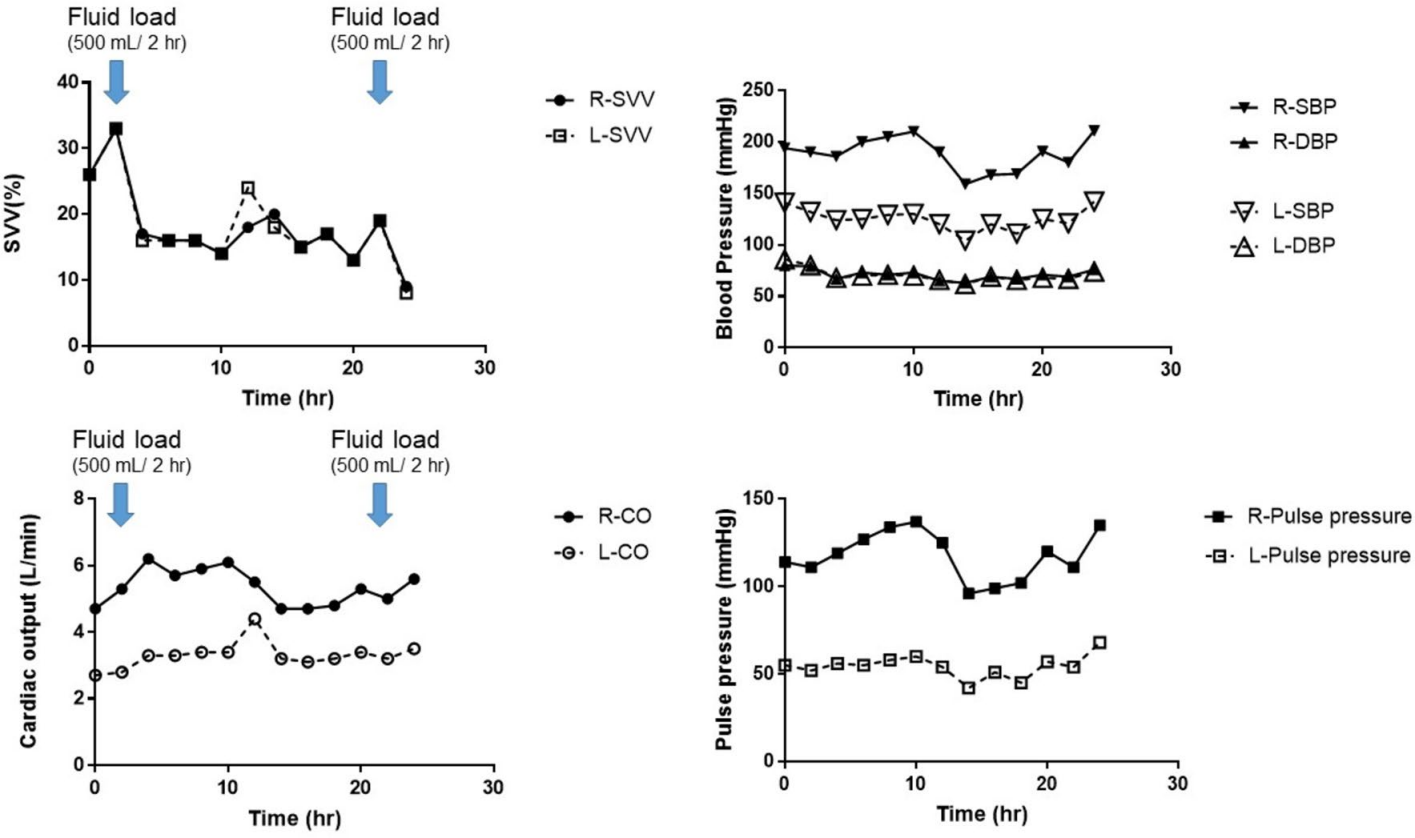
Changes in hemodynamic indicators over time. The data obtained from arterial pressure lines inserted into the right radial artery (healthy side) and the left radial artery (with proximal artery stenosis) were shown. The left radial artery pressure was about 50 mmHg lower than the right radial one. The pressure gradient was constant throughout, suggesting the presence of a substrate proximal stenosis. The cardiac output was also different between left and right, but both increased with infusion load. Left and right SVV remained almost constant, responding similarly to infusion load

## Data Availability

Not applicable.

## Abbreviations

CO: Cardiac output
ICU: Intensive care unit
SD: Standard deviation
SV: Stroke volume
SVV: Stroke volume variation
